# Khat Dependency and Psychophysical Symptoms among Chewers in Jazan Region, Kingdom of Saudi Arabia

**DOI:** 10.1155/2016/2642506

**Published:** 2016-02-28

**Authors:** Maged El-Setouhy, Rashad M. Alsanosy, Abdallah Alsharqi, Ahmed A. Ismail

**Affiliations:** ^1^Substance Abuse Research Center (SARC), Jazan University, Jazan, Saudi Arabia; ^2^Department of Community, Environmental and Occupational Medicine, Faculty of Medicine, Ain Shams University, Cairo, Egypt; ^3^Psychcare Center, Riyadh, Saudi Arabia; ^4^Occupational and Environmental Health Department, College of Public Health University of Iowa, Iowa City, IA, USA; ^5^Department of Community Medicine and Public Health, Faculty of Medicine, Menoufia University, Shebin El-Kom, Egypt

## Abstract

*Background*. Khat chewing is highly prevalent in Africa, Yemen and Jazan region, southwest of Saudi Arabia. Most of Jazani Khat chewers consider khat session as a social activity and do not consider khat dependency. The aim of this study was to explore khat dependency and its relationship with the psychophysical symptoms among chewers.* Methods*. Cross-sectional study on seventy Saudi male khat chewers living in Jazan area. Psychological dependence to khat chewing was evaluated using the Severity of Dependency Scale (SDS). The participants filled in a self-administrated assisted structured questionnaire designed to collect data about their medical history, neurological symptoms, and their chewing behavior.* Results*. Half (52.2%) of khat chewers showed psychological dependency. Those having longer khat sessions (≥6 hours) were more liable for dependency. Physical and psychological symptoms were more prevalent among khat dependent chewers.* Conclusions*. khat has a psychological dependence effect that can be measured by the SDS, even in low doses and with irregular use. SDS scale is a useful tool to expect the burden of either physical or psychological symptoms on khat chewers.

## 1. Introduction

Chewing and cultivation of khat are common in the East African countries and Arabian Peninsula [1]. In the Kingdom of Saudi Arabia, the habit is restricted to Southern area mainly in Jazan region [2]. Khat stimulant effect is attributed to its contents of cathinone, cathine, cathidine, Eduline, and ephedrine [3]. The most active component of khat is the cathinone, which is considered as a natural amphetamine. It helps chewers to improve their performance, stay alert, and initially increase their energy [4]. Its central nervous system (CNS) stimulant effect is about 7–10 times higher than cathine [5].

Khat physical and psychological hazards were controversially reported by many authors [1, 3, 4, 6–12]. The neurotoxic effects of khat were attributed to the release of dopamine from presynaptic storage sites [13]. Chronic khat use leads to a significant depletion of dopamine in several brain areas, particularly the nigrostriatal dopamine terminal projections [14]. Psychiatric effect of khat includes depression and psychosis among heavy chewers [15], psychosis precipitation in predisposed persons [4, 15], and symptom exacerbation in patients with preexisting psychiatric disorders [8].

Impaired concentration, insomnia, headache, migraine, mydriasis, impaired motor coordination, and fine tremors are also central nervous system deficits associated with khat use [7]. Cox and Rampes, 2003, reported in their review the behavioral effects of chewing khat that included euphoria, excitability, anxiety, irritability, hyperactivity, restlessness, and insomnia [4]. Khat chewers significantly complain of anorexia, insomnia, late wake-up next morning, and low work performance next day [8]. Anorexia was attributed to both the central amphetamine-like effect and delayed gastric emptying [16], while insomnia and delayed bedtime were expected to be due to the cathinone driven release of noradrenergic neurotransmitters [17].

Physical and psychological dependence of khat were reported by many authors [18–26]. Psychological dependence features include chewers urge to get their amount of khat on the expenses of their vital needs like food and mood changes during and after chewing [9, 20, 21, 27–29]. The Severity of Dependence Scale (SDS) developed by Gossop et al. [30] was used to evaluate psychological dependence to many abused drugs through collecting data about the dependence features [30–33]. SDS was validated and employed by Kassim and her colleagues to assess the psychological dependence of khat chewing among Yemeni population living in UK [18].

Features of physical dependence of khat chewing were also reported. Older and habitual khat chewers showed an increase in the amount of chewed khat [26, 34]. Other studies observed that khat chewers in their long sessions used to refresh the khat in the mouth [12, 35]. Some evidences also of withdrawal manifestations were found among khat chewers, who reported that they continued khat consumption to avoid unspecified symptoms and to avoid unpleasant feelings and depression [36]. Other withdrawal symptoms reported among khat quitters were lethargy, feeling sleepy, nightmares, feeling hot in the lower extremities, bad temper, and slight trembling [6, 7, 24, 37]. When Kassim and her colleagues [19] employed the criteria of dependence defined in the American Psychiatric Association Diagnostic Statistic Manual (DSM-IV, 1994) [38], they found that 31% of khat chewers reported dependence syndrome, 13% of them reported an increase in khat chewing, 19% reported cessation attempts, and 17% reported withdrawal symptoms including depression, increased appetite, and interrupted sleep.

We considered three main factors in creating this study: the first factor is the denial of khat dependence among Jazan khat chewers: they consider it merely as a social gathering substance like tea and coffee [1, 37] although quitters expressed withdrawal symptoms [37]; second, khat cultivation, trafficking, selling, and use are prohibited in Saudi Arabia which forces chewers to use it on irregular bases and with a lesser amount than those living in countries where khat is legal [37, 39]. The third factor was the absence of khat dependency studies in Jazan region where khat chewing is highly prevalent, especially among males.

We aimed in this study to measure dependence among khat chewers using SDS and study the risk of complaining of physical and psychological symptoms among chewers according to their dependence scores as a first time evaluation of the SDS in Saudi Arabia.

## 2. Subjects and Methods

### 2.1. Sitting

A cross-sectional study was conducted in Jazan region, which is located at the far southwest of Saudi Arabia (adjacent to Yemen) during the period from October 2011 to May 2012.

### 2.2. Participants

Seventy male khat chewers were recruited by local advertising in the public places, for example, restaurants, mosques, and commercial shopping centers. Selecting participants and used methodology were described in detail elsewhere [40]. In brief, adult chewers from Jazan region were included in the study. Chewers at least once weekly and for a year are included in this study. Our study physicians reviewed the participants' medical history and examined them clinically to exclude those with peripheral neuropathy, previous head or back injuries, or those abusing or addictive to other drugs. We paid each participant a hundred Saudi Riyals (approximately USD $27.00) to cover transportation expenses and time required for the study. The research protocol was approved by IRB at Jazan University, Saudi Arabia. The consent form was explicitly detailed to all participants before signing it and before participating in the study.

### 2.3. Study Tool

All participants filled in a self-administrated assisted structured Arabic questionnaire of four main sections. First section was to collect the demographic data. Second one was reporting the relevant medical history. The third section was evaluating the neurologic symptoms. It was developed from the Q16 questionnaire [41]. The symptoms were reported on a 5-digit scale: never, once, 1/month, 1/week, and more than once/week. The fourth section was the Severity of Dependence Scale (SDS) of khat adopted by Kassim and her colleagues to measure khat dependence of Yemeni living in UK [18]. It consists of five items that measure dependence over the last 12 months. It uses four scale scores for four items: “never or almost never,” “sometimes,” “often,” and “always or nearly always,” and “not difficult,” “quite difficult,” “very difficult,” and “impossible” for one item. Scores for each item range from 0 to 3 and the total score ranges from 0 to 15. In the present study, a cutoff point of four was used as suggested by Topp and Mattick, 1997 [42].

### 2.4. Statistical Analysis

Data was double entered using MS Access and analyzed with SPSS (Statistical Package for Social Sciences) software version 17. Chi-square test was used to examine the differences between percentages. The software was also used to draw histogram for SDS scores distribution among khat chewers. Depending on the common reporting of symptoms among participants; reporting was recategorized into two categories, frequent reporting, which includes 1/month, 1/week, and more than once/week, and infrequent which includes never and once. Logistic regression model was adopted to control for the confounders that may affect khat chewing dependency. In addition to dependency case on the SDS, age, smoking, chewing dose, and duration were included in the model to account for its impact.

## 3. Results

### 3.1. Participant Characteristics

Our study participants had been chewing khat for an average of 7 years. They reported that they chew khat approximately 3 days/week, for an average of 5 hours/session. They chew between 1/4 of a bundle to a complete bundle/session. Almost half of them (57%) chew half a bundle of khat per sitting (Table 1).

### 3.2. Severity of Dependence Scale (SDS) on Khat

**Figure 1:**
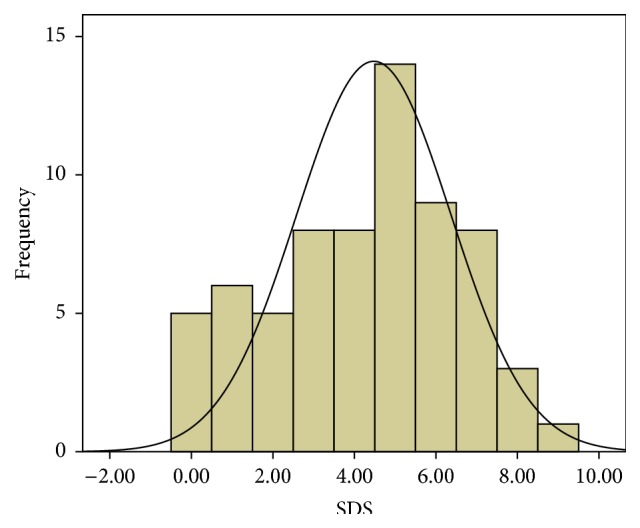
Frequency distribution of SDS scores among the khat chewers.

67 out of the seventy chewers completed the SDS scale. Considering a cutoff point of four, 32 (47.8%) of chewers were nondependent, while 35 (52.2%) were dependent. The mean score of khat chewers was 4.3 ± 2.3 (Figure 1).

### 3.3. Relationship between SDS and Chewing Behaviors

Although dependent khat chewers showed higher use of khat, the only significant behavior related to dependency was the length of the khat sessions (*p* = 0.03, Table 2).

### 3.4. Physical and Psychological Symptoms among Chewers according to SDS

Physical and psychological symptoms were more frequent among dependent chewers than nondependents.  Table 3 shows that the frequency of half of the symptoms (12 from a total of 24) was significantly higher among dependent than nondependent ones (*p* < 0.05). These results were gotten after controlling of other confounders that may predispose to these symptoms: age, smoking, years of chewing, amount of chewed khat, and duration of chewing session. The risk of having those significant symptoms among dependent chewers ranged from 3 to 15.8 (last column, Table 3).

## 4. Discussion

Our study is the first to evaluate kaht dependence in one of its original places of khat cultivation and chewing. Chewing prevalence among Jazan College and secondary schools students was estimated to be 21.4 [1]. A cutoff point for the SDS scale of 4 was used as recommended by Topp and Mattick [42] which is lower than 5 that was used by Kassim and her colleagues for the Yemeni chewers in UK [18]. However, the interpretation of our SDS results revealed that around half of our chewers (52.2%) were dependent, which is very close to the percent of dependents among Yemeni chewers (51.0%) living in UK [18]. The mean SDS score of our chewers was 4.3 (Figure 1), while it was 5.5 in Kassim et al. study. This would be explained by the chewing behavior of our chewers, where the majority of them (57.1%) chew half of a bundle during the chewing session, only half of them chew more than 3 days per week, and most of them (85.7%) spent less than 6 hours in each chewing session (Table 2) which is less than that of Kassim et al. study. However, the SDS results of our study are still compatible with those of other studies that indicate the psychological dependence of chewing khat [20, 21, 27]. The most related khat chewing feature to SDS was the duration of chewing session, where more dependent chewers (20.6%) spent more than 6 hours in a chewing session (Table 2). This confirms the psychological dependence nature of khat chewing habit.

We evaluated a wide range of physical and psychological symptoms in relation to the psychological dependence to khat. The symptom questionnaire that has been developed form Q16 questionnaire [41] showed that high percentages of dependent Jazan khat chewers were complaining of physical and psychological symptoms. Dependent khat chewers reported frequently more physical and psychological symptoms of dizziness, feeling of anxiousness and irritability, nausea and vomiting, difficulty of seeing at night, being absentminded, headache, loss of appetite, fast heart rate, difficulty with balance and coordination, blurred vision, difficulty in concentration, and numbness than nondependent chewers (Table 3). These reported symptoms constitute most of the neurological functions: autonomic (nausea, vomiting, excessive sweating, loss of appetite, and fast heart rate), motor (difficulty with balance, weakness in arms and legs, and involuntary movements), cognitive (difficulty in concentration), behavioral (feeling irritable and depressed), and other nonspecific symptoms (headache, dizziness, and difficulty in falling asleep), which gives a good indicator of the effect of khat chewing on the neurological system performance. The risk of complaining of these symptoms among dependent chewers ranged from 3 times (loss of appetite and difficulty in balance) to about 16 times (difficulty seeing at night).

Although most of our participants were using relatively small amounts of khat/session, they still show manifestations of dependency similar to Yamani chewers who used a complete bundle for three successive days [8]. Another study on Yamani khat chewers had more anxiety, irritability, and insomnia symptoms [4]. Similar results were observed in the studies of neuropsychiatric disorders of chewing khat with different amounts [9, 15, 27]. The effect of khat components on the central nervous system would be augmented to cause leukoencephalopathy [10]. Animal studies also supported our results, as it was observed that administration of khat extract to lab animals leads to tremors and stereotyped motor activity at low doses and seizures at high doses [43], enhanced the baseline aggressiveness of isolated rats [14], and precipitated behavioral sensitization for motor activity [44].

A prospective cohort study is needed to follow up chewers for a period of time to record the start of the appearance of physical and psychological symptoms and relate it to khat chewing. Measuring the levels of biomarkers (dopamine or serotonin and/or exposure biomarkers (cathinone and its metabolites) is also needed to correlate the physical and psychological symptoms with these biological indices. Clear definitions of khat use behavior (regular, beginners, etc.) as well as chewing levels (heavy, moderate, and light) would be measured according to the psychological features but with larger sample size.

## 5. Conclusions

We conclude from the study that khat chewing has a psychological dependence effect even in low doses and with irregular use. This psychological dependency still can be measured by the SDS and confirmed by the reporting of more physical and psychological symptoms by the dependent group. Outcomes of the study indicate the neuropsychological drawbacks of chewing khat and the importance of SDS and neurological evaluation in measuring this impact.

## Figures and Tables

**Table 1:** Sociodemographic characteristics of the study participants.

	Khat chewers (*n* = 70)
	Number (%)
Age (years)^*∗*^	25 (5.2)
Years of education^*∗*^	12 (3.0)
Occupation	
Employee	17 (24.3)
Student	39 (55.7)
Does not work	14 (20.0)
Marital status	
Married	12 (17.1)
Single	58 (82.9)
Smoking	
No	25 (35.7)
Yes	45 (64.3)
Amount of khat used/session	
1/4 of a bundle	19 (27.1)
1/2 of a bundle	40 (57.1)
A bundle	11 (15.7)
Number of chewing days/week^*∗*^	3 (2)
Chewing years^*∗*^	7.5 (5.6)
Duration of the session in hours^*∗*^	5 (2)
Range	2–16

^*∗*^Mean and SD were reported.

**Table 2:** Relationship between SDS and chewing features.

	SDS		Chi-square *p* value	OR (CI)
	Nondependent (*N* = 32) *N* (%)	Dependent (*N* = 35) *N* (%)		
Years of chewing				
<10	24 (75.0)	25 (73.5)	0.9	1.1 (0.4–3.3)
≥10	8 (25.0)	9 (26.5)		
Number of days chewing khat/week				
1-2	17 (53.1)	15 (44.1)	0.5	1.4 (0.5–3.8)
≥3	15 (46.9)	19 (55.9)		
Duration of the session (hours)				
<6	31 (96.9)	27 (79.4)	0.03^*∗*^	8.0 (1.0–96.6)
≥6	1 (3.1)	7 (20.6)		
Amount of chewed khat (bundles)				
1/4	10 (31.2)	9 (26.5)	0.7	1.3 (0.4–3.7)
1/2 or more	22 (68.8)	25 (73.5)		

^*∗*^Significant *p* value; Fisher exact test was used.

**Table 3:** Risk of physical and psychological symptoms among khat users according to the SDS.

	SDS		*p* value	OR (CI)
	Nondependent (*N* = 32) *N* (%)	Dependent (*N* = 35) *N* (%)		
Dizziness	5 (15.6)	26 (45.7)	0.005^*∗*^	6.5 (1.7–23.6)
Feeling anxious	10 (31.3)	20 (57.1)	0.02^*∗*^	3.5 (1.2–10.0)
Nausea & vomiting	7 (21.9)	20 (57.1)	0.003^*∗*^	5.9 (1.8–18.9)
Feeling tired	15 (46.9)	21 (60.0)	0.21	1.9 (0.7–5.2)
Excessive sweating	12 (37.5)	16 (45.7)	0.4	1.6 (0.6–4.3)
Difficulty seeing at night	2 (6.3)	14 (40.0)	0.002^*∗*^	15.8 (2.7–91.4)
Being absentminded	6 (18.8)	18 (51.4)	0.007^*∗*^	5.1 (1.6–16.4)
Headache	12 (37.5)	23 (65.7)	0.03^*∗*^	3.1 (1.1–8.6)
Loss of appetite	16 (50.0)	25 (71.4)	0.04	3.0 (1.1–8.8)
Fast heart rate	9 (28.1)	17 (48.6)	0.06	2.7 (0.9–7.7)
Difficulty with balance	8 (25.0)	16 (45.7)	0.048	3.0 (1.0–9.0)
Blurred vision	4 (12.5)	15 (42.9)	0.006	5.9 (1.6–4.7)
Difficulty in concentration	6 (18.8)	17 (48.6)	0.005^*∗*^	5.7 (1.7–19.7)
Numbness	6 (18.8)	14 (40.0)	0.03	3.9 (1.1–13.4)
Momentary loss of consciousness	5 (15.6)	9 (25.7)	0.16	2.7 (0.7–10.7)
Feeling irritable	12 (37.5)	15 (42.9)	0.5	1.5 (0.5–4.2)
Shaking or trembling of hands	9 (28.1)	9 (25.7)	0.8	0.9 (0.3–2.9)
Difficulty in falling asleep	15 (46.9)	14 (40.0)	0.6	0.8 (0.3–2.1)
Weakness in arms & legs	6 (18.8)	10 (28.6)	0.4	1.7 (0.5–5.5)
Change in smell & taste	6 (18.8)	11 (31.4)	0.1	2.5 (0.7–8.6)
Feeling depressed	7 (21.9)	13 (37.1)	0.1	2.3 (0.7–6.8)
Excessive salivation	11 (34.4)	10 (28.6)	0.7	0.8 (0.3–2.4)
Involuntary movements	7 (21.9)	12 (34.3)	0.2	2.1 (0.7–6.5)
Tinnitus	7 (21.9)	12 (34.3)	0.2	2.3 (0.7–7.5)

Odds ratios were adjusted in the logistic regression model for age, smoking, years of chewing, amount of chewing, and duration of chewing session.

## Abbreviations

SDS: Severity of Dependency Scale
CNS: Central nervous system
SPSS: Statistical Package for Social Sciences.
